# The IL-23/IL-17 axis in Behçet’s syndrome pathogenesis: from immunological perspectives to therapeutic implications

**DOI:** 10.3389/fimmu.2026.1761519

**Published:** 2026-03-10

**Authors:** Mohammad E. Naffaa, Fadi Hassan, Mahmud Omar, Kerem Abacar, Dennis McGonagle

**Affiliations:** 1 Rheumatology Unit, Galilee Medical Center, Nahariya, Israel; 2 Azrieli’s Faculty of Medicine, Bar-Ilan University, Safed, Israel; 3 Maccabi Healthcare Services, Tel-Aviv, Israel; 4 Sackler Faculty of Medicine, Tel-Aviv University, Tel-Aviv, Israel; 5 Leeds Institute of Rheumatic and Musculoskeletal Medicine, National Institute for Health and Care Research (NIHR) Research Center, University of Leeds, Leeds, United Kingdom

**Keywords:** Behcet’s syndrome, interleukin 17, interleukin 23, MHC-1-opathy, pathogenesis

## Abstract

Behçet’s Syndrome (BS) is a systemic vasculitis characterized by variable vessel involvement and an elusive etiology, though immunogenetic studies strongly implicate the IL-23/IL-17 axis which bridges innate and adaptive immunity, orchestrating type 17 T-cell responses thus modulating neutrophil function- with this cell a central player in both BS clinical features and immunopathology. Additionally, the contribution of Th1 cytokines—such as interferon gamma (IFNγ) and tumor necrosis factor alpha (TNFα)—reflects the broader immune plasticity observed in BS pathophysiology. Despite the immunogenetics incriminating the IL-23/IL-17 axis, clinical evidence confirming the role of IL-23/IL-17/inhibition in BS therapy is still limited including disappointing results with secukinumab in trials for Behçet’s uveitis. However, emerging evidence from small-scale retrospective studies, prospective trials, and case reports indicates that IL-23/IL-17 axis inhibition may benefit mucocutaneous and articular manifestations, as well as neuro-Behçet’s disease and the licensed PDE4 inhibitor apremilast regulates multiple aspects of IL-23/17 axis and neutrophil biology. Interestingly, anti-IL-17 therapy has been linked to BS induction. Herein, we discuss IL-23/IL-17 axis inhibition in BS and why it should be used cautiously and be limited to mucocutaneous and/or articular manifestations at this juncture. Further randomized controlled trials are imperative to dissect the IL-23/IL-17 axis in BS including high-dose anti-IL-23 therapy antagonism given that neutrophils are an abundant source of IL-23 and consider novel strategies including IL-23R antagonism.

## Introduction

Behçet’s syndrome (BS) is a systemic vasculitis mainly affecting the venous tree but can also affect all types and sizes of blood vessels (1–3). Oral and genital ulcers are characteristic of BS with other manifestations such as ophthalmic, vascular and neurologic that can be organ or even life-threatening (1–3). The current treatment guidelines of BS advocate an organ or involvement-based approach, rather than empiric therapy (4, 5). Although the introduction of anti-tumor necrosis factor alpha inhibitors (anti-TNFα) has revolutionized the treatment of several BS manifestations, especially ocular and gastrointestinal (6–18), many patients still have treatment-refractory manifestations emphasizing the need for new additional therapeutic options for the BS therapeutic arsenal (1, 4). It is anticipated that novel therapy development will emerge from an improved etiopathogenic understanding of disease immunopathogenesis.

The etiology of BS remains incompletely understood but it is believed that environmental factors (such as infections) or self-antigens and genetic susceptibility activate the adaptive immune system that supervenes on top of innate immune system activation (1). Human leukocyte antigen B*51 (HLA-B*51) represents the strongest genetic risk factor, but additional single-nucleotide polymorphisms (SNPs) have also been identified, particularly in IL10, Endoplasmic reticulum aminopeptidase 1 (ERAP1), and the IL23R–IL12RB2 locus (19, 20). Indeed, BS shares several clinical and immunogenetic features with spondyloarthropathies (SpA) spectrum disorders such as ankylosing spondylitis, psoriatic arthropathy, psoriasis and inflammatory bowel disease (21). Moreover, the term “MHC-1-opathy” was suggested as a unifying concept for these conditions including BS due to the either proven or putative involvement of CD8 T-cells and common SNPs in the IL-23R, SNPs in the related IL-23 cytokine genes and related Janus Kinase (JAK) signaling pointing to central involvement of the IL-23/17 axis (21).

The aforementioned immunogenetics broadly equate with the therapeutic efficacy of both IL-23 and IL-17 inhibition across SpA spectrum inflammation, albeit with nuanced differences between the gut and spine with IL-23 and IL-17 inhibition (22–24). These insights naturally lead to extrapolations and considerations of whether the IL23/17 axis represents a novel therapeutic target to address the unmet needs in BS. The existing therapeutic options include mainly anti-TNFα and phosphodiesterase 4 (PDE-4) inhibitors with the largest amount of evidence, but also IL-12/23 (p40) inhibitors, IL-23 (p19) inhibitors, JAK inhibitors and IL-17 inhibitors (1). Recently, further evidence has also emerged for the comparative effectiveness of type 1 interferon (IFN) therapy versus infliximab in BS (25). Of note, there is a compelling body of evidence for PDE-4 inhibitor mechanism of action in neutrophil function suppression, which is of considerable relevance given that neutrophils are significant producers of the IL-23 cytokine and are clinically and microscopically evident in BS-associated pathology (26).

This paper critically reviews the role of IL-23/17 axis in the pathogenesis of BS, presents the available clinical trial data, and summarizes the reported paradoxical cases where anti-IL-17 inhibition induced a Behçet’s-like syndrome. We use this as a platform to consider the way forward for IL-23/17 axis inhibition in BS and where could find clinical application.

## Pointers to the role of IL-23/17 axis in BS pathogenesis

### Immunogenetics

#### Genetic variants in the IL-23 pathway

Genome-wide association studies (GWAS) have identified multiple SNPs in the IL23R gene that are associated with increased susceptibility to BS. For example, the rs17375018 SNP has been linked to BS risk in the Turkish population, while the rs11209026G allele is more frequent in male patients, and rs7517847G is enriched in individuals with genital ulcers (27, 28). Additionally, the GG+GT genotypes of *IL23R* rs1884444 were found to contribute to intestinal BS in Korean patients, and when combined with the *IL17A* rs8193036 TT genotype, further increased the risk of BS compared to individuals with no-risk or single-risk genotypes (29, 30). Moreover, increased gene copy numbers of *IL23A* were reported in a large-scale study to be associated with BS (31).

#### Genetic variants in the IL-17 pathway

**Figure 1:**
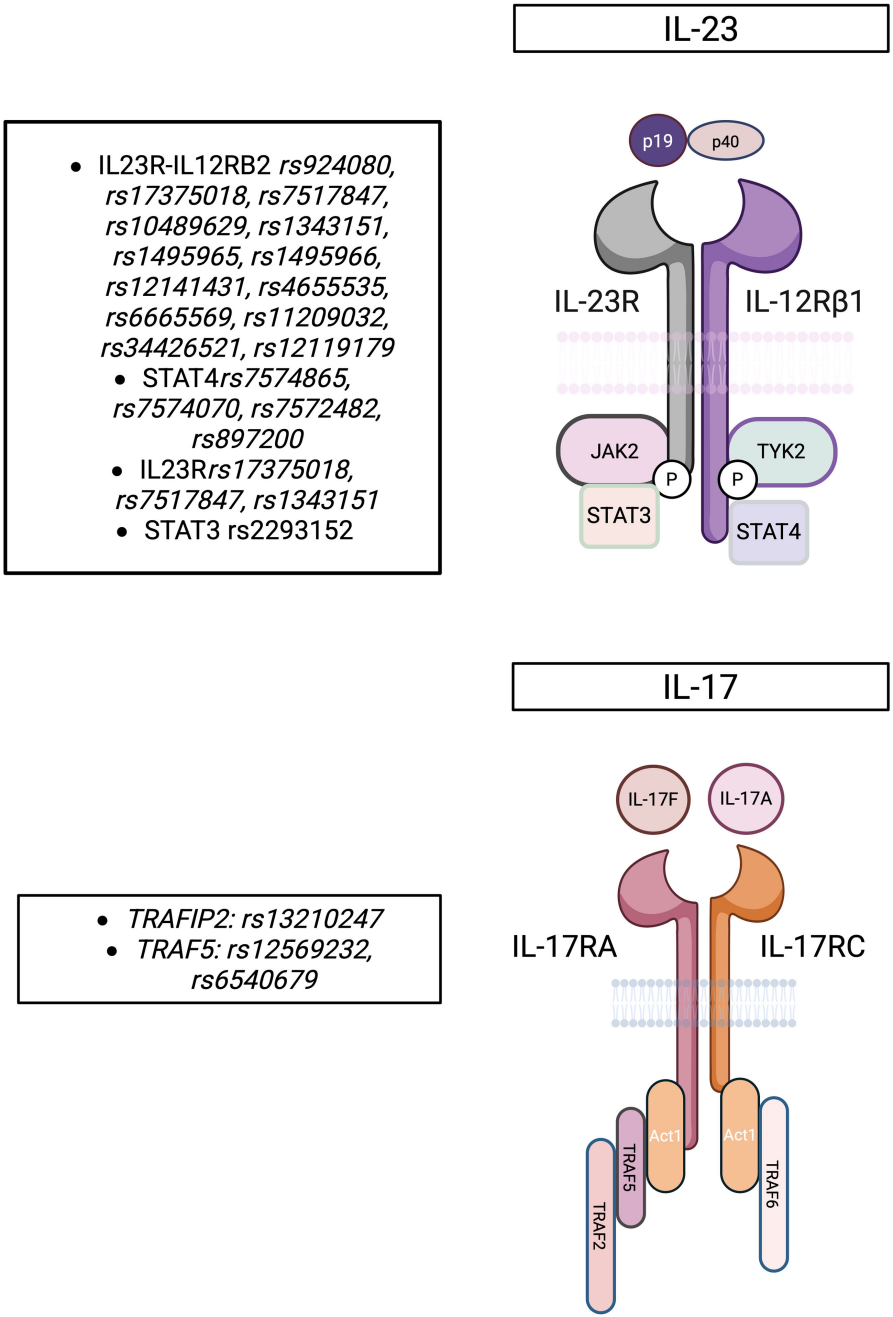
SNPs associated with Behçet Syndrome affecting the IL-23/IL-17 cytokine pathway. This figure illustrates the IL-23 and IL-17 signaling pathways and their associated SNPs linked to Behçet’s syndrome (BS). IL-23 signals through IL-23R/IL-12Rβ1, activating JAK-STAT pathways to drive Th17 responses. IL-17A/F binds IL-17RA/IL-17RC, triggering downstream signaling via TRAF2, TRAF5, and TRAF6. Key SNPs in IL23R, IL12RB2, STAT3, and TRAF genes may influence receptor function, cytokine signaling, and immune dysregulation in BS.

Several studies have implicated IL-17-related genes in BS pathogenesis. The TT genotype of *IL17A* rs8193036 was linked to intestinal BS in Korean patients (29), and a separate Korean study found significant differences in the A126G SNP of *IL17F* between BS patients and healthy controls (30). A Turkish case-control study revealed associations between BS susceptibility and multiple variants: *IL17A* rs8193036 (C/T), rs3819025 (G/A), rs3804513 (A/T); *IL17F* rs2397084 (T/C); and *IL17RC* rs708567 (C/T) (32). In addition, a recent meta-analysis confirmed strong associations between *IL17A* rs4711998 and rs8193036 with disease progression in BS, reinforcing their immunopathogenetic relevance (33). **Figure 1** summarizes the main SNPs associated with BS that affect the IL-23 and IL-17 cytokine pathways.

### Cellular immunology- T-cells

Studies have highlighted the pivotal role of Th17 (CD4 T cells producing IL-17) and Tc17 (CD8 T cells producing IL-17) cells in the pathogenesis of BS as evidenced by their presence not only in the peripheral blood but also in affected tissues including skin, gastrointestinal tract, cerebrospinal fluid and brain parenchyma (34–38). IL-23 secreted by activated neutrophils promotes the differentiation of CD4 T cells into Th1 and Th17 cells with subsequent secretions of interferon gamma (IFNγ), IL-17 and IL-23 (39, 40). At this point, it should be emphasized that Rosine and colleagues showed that among patients with axial SpA, neutrophils were not a source of IL-17 production even after strong stimulation (41). Further evidence for the importance of Th17 cells in the pathogenesis of BS has been provided by Kim and colleagues as they demonstrated a higher ratio of Th17/Th1 in blood among BS patients compared to healthy controls, mainly in patients with uveitis or folliculitis (35).

Regulatory T cells (Treg) are a subset of T cells that modulate excessive effector T cell activation through the production of TGFβ and IL-10 (42). Both dysregulated Th17 and Treg cell functions have been reported in patients with BS, and Th17/Treg imbalance, represented by an increase in Th17 and a decrease in Treg functions, has been suggested to contribute to disease pathogenesis (43). Direskeneli et al. reported an antigen-driven oligoclonal increase of T cells in BS, both CD4 and CD8 T cells (44). CD8 T cells are an increasingly appreciated source of IL-17 production, a finding that is particularly relevant in MHC-1-associated disorders. BS patients with active disease who are HLA-B*51 positive exhibit a higher proportion of CD8 T cells compared to healthy individuals and inactive BS (45).

**Figure 2:**
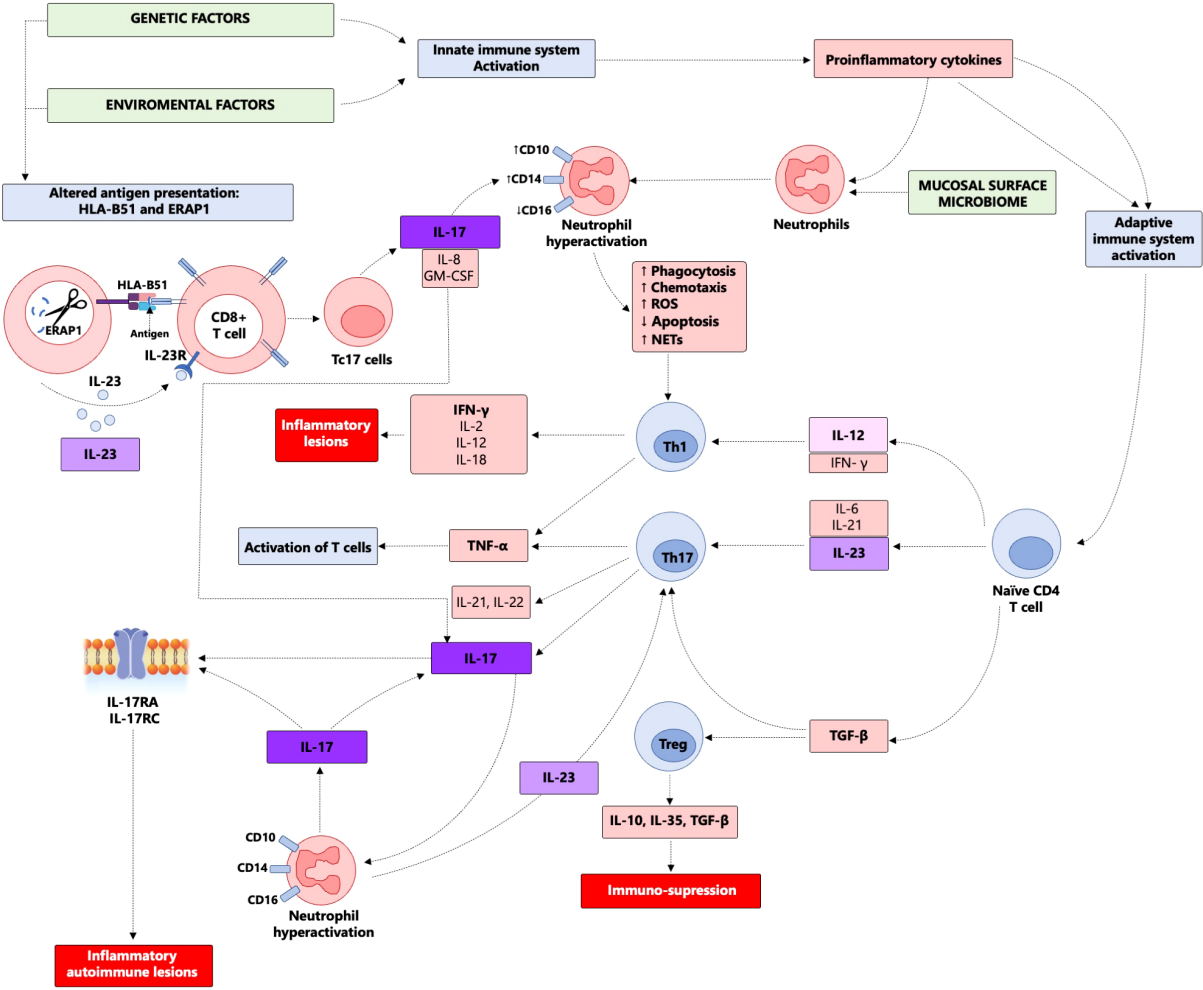
Pathogenesis of Behcet’s syndrome. This figure illustrates Behcet’s syndrome pathogenesis which involves genetic factors like HLA-B51 and ERAP, influencing CD8+ T cell activation and IL-17 production. IL-23 drives Th17 differentiation and further IL-17 release. Neutrophils are central effectors, amplifying inflammation via IL-23 and IL-17 pathways and NETosis. Imbalances in the IL-23/IL-17 axis contribute to tissue damage.

It has been reported that abnormal peptide trimming by ERAP1 in the context of HLA B*51 positivity drives the activation and differentiation of the CD8 T cells into Tc17 cells that secrete different cytokines including IL-17, IL-8, and granulocyte–macrophage colony-stimulating factor (GM-CSF), which have diverse effects, such as enhancing neutrophil activation and facilitating the interaction between innate and adaptive immunity in BS immunopathogenesis (21). CD8 T-cell numbers were elevated in the aqueous humor of BS patients with ocular involvement (36). Studies also indicate that in BS skin lesions, IL-17-secreting T cells predominantly originate from CD8 T cells (now designated Tc17 cells) rather than CD4 cells (the classical Th17 CD4 T cells) (37). Collectively, these studies support a role for IL-17 CD8 T-cells especially since such cytokine elaboration is known to depend on IL-23R signaling in general and also in BS (**Figure 2**).

### Cellular immunology- neutrophils

**Figure 3:**
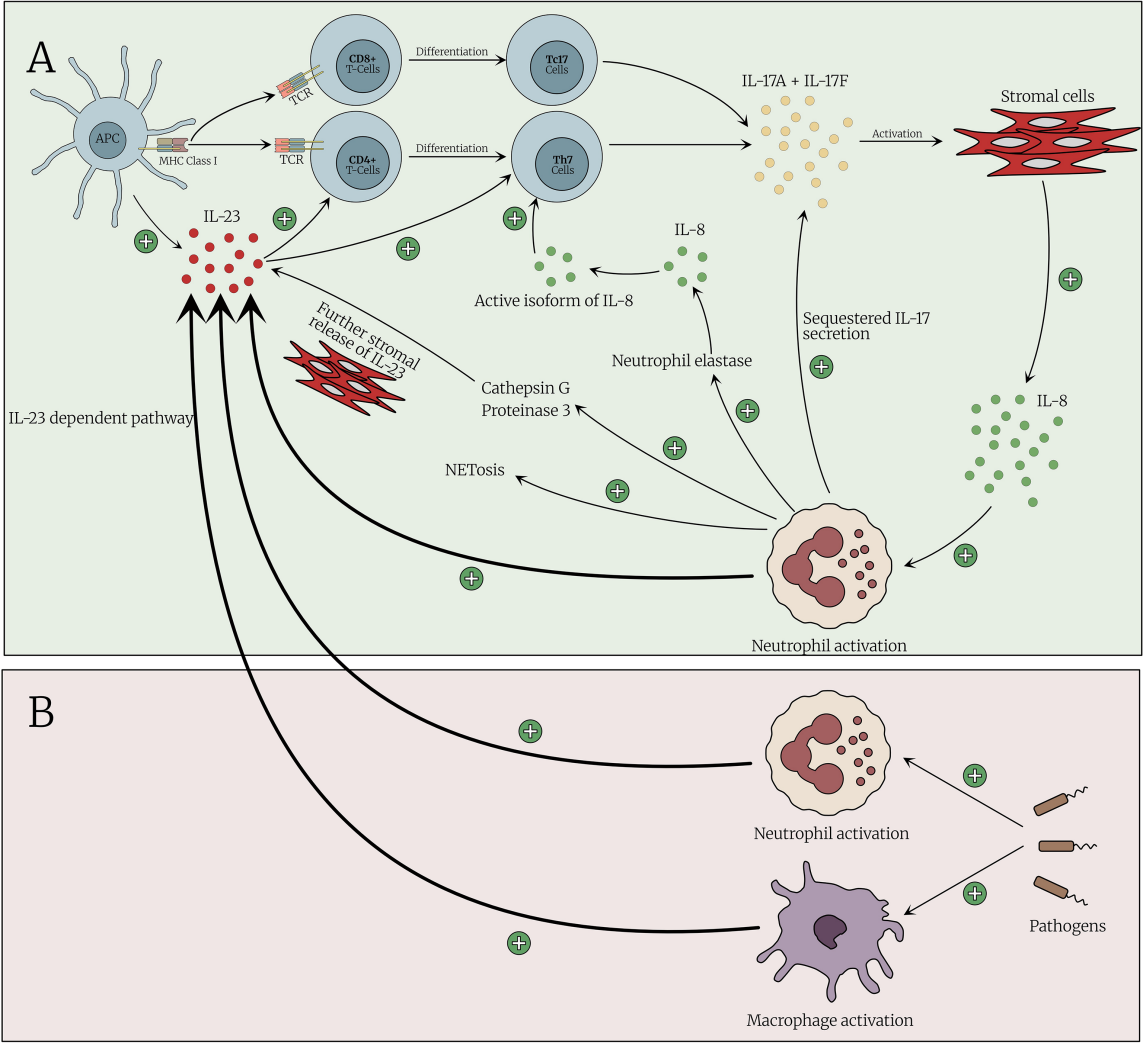
The cross talk between IL-23/17 axis and neutrophils activation. **(A)** The pathway is initiated by an antigen-presenting cell (APC) that presents an antigen to naïve CD4^+^ T cell. Antigen presentation induces the secretion of IL-23, which drives the immune response toward the differentiation into Type 17 T cells. These activated cells secrete IL-17A and IL-17F, leading to activation of stromal cells to produce IL-8. IL-8 acts, among other targets, on neutrophils, leading to their activation. Activated neutrophils further secrete IL-23 reinforcing the Th17 immune response. Additionally, neutrophil activation enhances NETosis and the release of proteases, further amplifying this response. **(B)** Emerging evidence suggests that neutrophils, and macrophages, may also initiate the Th17 immune response, rather than solely acting downstream. Upon encountering pathogens, neutrophils can release of IL-23,either directly or indirectly, thus promoting a Th17-skewed immune profile.

Neutrophils are the cell type that dominates the clinical landscape of BS with pustular lesions clinically and histologically. Neutrophils are a major source of pro-inflammatory cytokines, such as IL-23, that are involved in the differentiation of CD4 T cells into T helper 17 (Th17) and T helper 1 (Th1). Neutrophils drive skeletal inflammation in the IL-23 dependent SKG mouse model. Stavre et al. considered the role of neutrophils in the experimental SKG mouse model of SpA and in human axial entheses and showed that neutrophils with inducible IL-23 production are prominent in early murine SpA-related enthesitis (40). Kvedaraite and colleagues demonstrated that tissue-infiltrating neutrophils were the main source of IL-23 in the colon of pediatric patients with inflammatory bowel disease (IBD) (39). Activated by proinflammatory cytokines (e.g. IL-17), neutrophils actively participate in the systemic inflammatory response at several levels: IL-23 secretion with subsequent augmentation of IL-17 secretion, and the production of reactive oxygen species (ROS) and neutrophil extracellular traps (NETs) leading to endothelial injury and activation and ultimately to thrombus formation. Thus, rather than merely being terminal effectors, neutrophils may drive complex positive feedback loops to regulate the adaptive immune response in BS (**Figure 3**).

Neutrophilic inflammation has been demonstrated in several lesions of BS patients, including skin, mucosal, synovial, gastrointestinal, vascular and pathergy lesions (46–51). Neutrophils in BS have been reported to be hyperactivated relative to healthy controls. Bettiol and colleagues showed that patients with active (non-vascular) BS had remarkably increased NET levels compared to patients with inactive disease and controls. They also demonstrated that colchicine significantly reduced oxidation-induced NET production and cell apoptosis in isolated neutrophils (52). Interestingly, Novak et al. reported that enzymatically active neutrophil elastase, coupled with reduced secretory leucocyte protease inhibitor (SLPI), an endogenous inhibitor, in the saliva of BS patients may contribute to the development of recurrent oral ulcerations in BS patients (53).

Furthermore, neutrophil activation and enhanced ROS production were associated with altered fibrinogen structure and impaired fibrinogen function in BS patients, that might contribute to thrombosis (54). Le Joncour and colleagues showed that activation surface markers (CD64, CD66b, CD11b, and CD11c), ROS production, and NETosis were up-regulated in BS patients’ neutrophils compared to healthy donor neutrophils. Additionally, they showed that inhibition of PDE4 by apremilast strongly inhibited neutrophil surface activation markers as well as ROS production and NETosis (55). Indeed, the effect of PDE4 inhibition on neutrophils has already been reported in the context of pulmonary diseases such as chronic obstructive pulmonary disease (COPD) (56), and acute respiratory distress syndrome (57). Kim et al. demonstrated that the PDE4 inhibitor, roflumilast, attenuated histopathological changes associated with lipopolysaccharide (LPS)-induced lung injury and inhibited the accumulation of neutrophils in bronchoalveolar lavage fluids in LPS-induced acute lung injury (ALI) during neutropenia recovery in murine model (58). Furthermore, roflumilast was shown to inhibits neutrophil chemotaxis directly via cAMP-mediated mechanism in COPD (59). Interestingly, roflumilast N-oxide, the active metabolite of roflumilast, resulted in significant impairment of polymorphonuclear cells (PMN) adhesion to activated platelets and mediated NET release by PMNs adherent on fibrinogen-coated surfaces (60).

Li et al. demonstrated that excessive BS NETs promoted macrophage activation and facilitated IFN-γ^+^ CD4 T cells differentiation (61). Altogether, these findings emphasized the potential effect of PDE4 inhibition on neutrophil activation, migration and participation in the inflammatory and thrombotic process.

### Cytokine immunology

IL-23, a heterodimeric cytokine produced by activated macrophages and dendritic cells, plays a crucial role in BS pathogenesis (62). While sharing the p40 subunit with IL-12, its unique p19 subunit confers distinct functions. IL-23 promotes differentiation and expansion of CD4 T cells into Th17 cells producing IL-17 (62). IL-17, in turn, stimulates the production of several chemokines and cytokines, including TNF-α, IL-6, and GM-CSF, leading to neutrophil recruitment and tissue inflammation (63). The neutrophils recruited and activated at disease sites by T cells and IL-17, in turn, can promote the development of Th17 and Tc17 T cells (63, 64). Multiple studies have demonstrated upregulation of IL-23 cytokine and its receptor in BS patients (65, 66). Notably, IL-23 levels correlate with BS activity and sustained elevations in Th17 cell counts (65, 67–69). Wakiya and colleagues showed that following apremilast administration, the TNF-α and IL-23 levels significantly decreased in concordance with clinical improvement (70). Another study, by Gheita et al, reported that serum IL-23 level was significantly higher in patients with BS compared to controls and the significantly increased serum IL-23 correlated with disease activity (71). However, we must emphasize that serum IL-23 level measurement exceeded 100 pg/ml, representing extremely elevated levels compared to other IL-23R associated conditions and the BS studies needs further confirmation.

IL-17 is produced by various immune cells, including, but no limited to, Th17 cells, Tc17 cells, γδ T cells and NK cells (72). The IL-17 family consists of six members: IL-17A, IL-17B, IL-17C, IL-17D, IL-17E, IL-17F. The latter, IL-17F exhibits the highest homology with IL-17A, and they can form heterodimers, whereas the other IL-17 family members function as homodimers when binding to their respective receptors (73). Jadideslam and colleagues showed that IL-17 mRNA expression and serum levels were significantly higher in the BS patients compared to healthy controls, and interestingly, they also showed that IL-17 were higher among patients with inactive BS uveitis compared to patients with active uveitis (74). Na and colleagues demonstrated that up-regulated IL-17 expression may be associated with clinical activity of BS (69).

**Figure 4:**
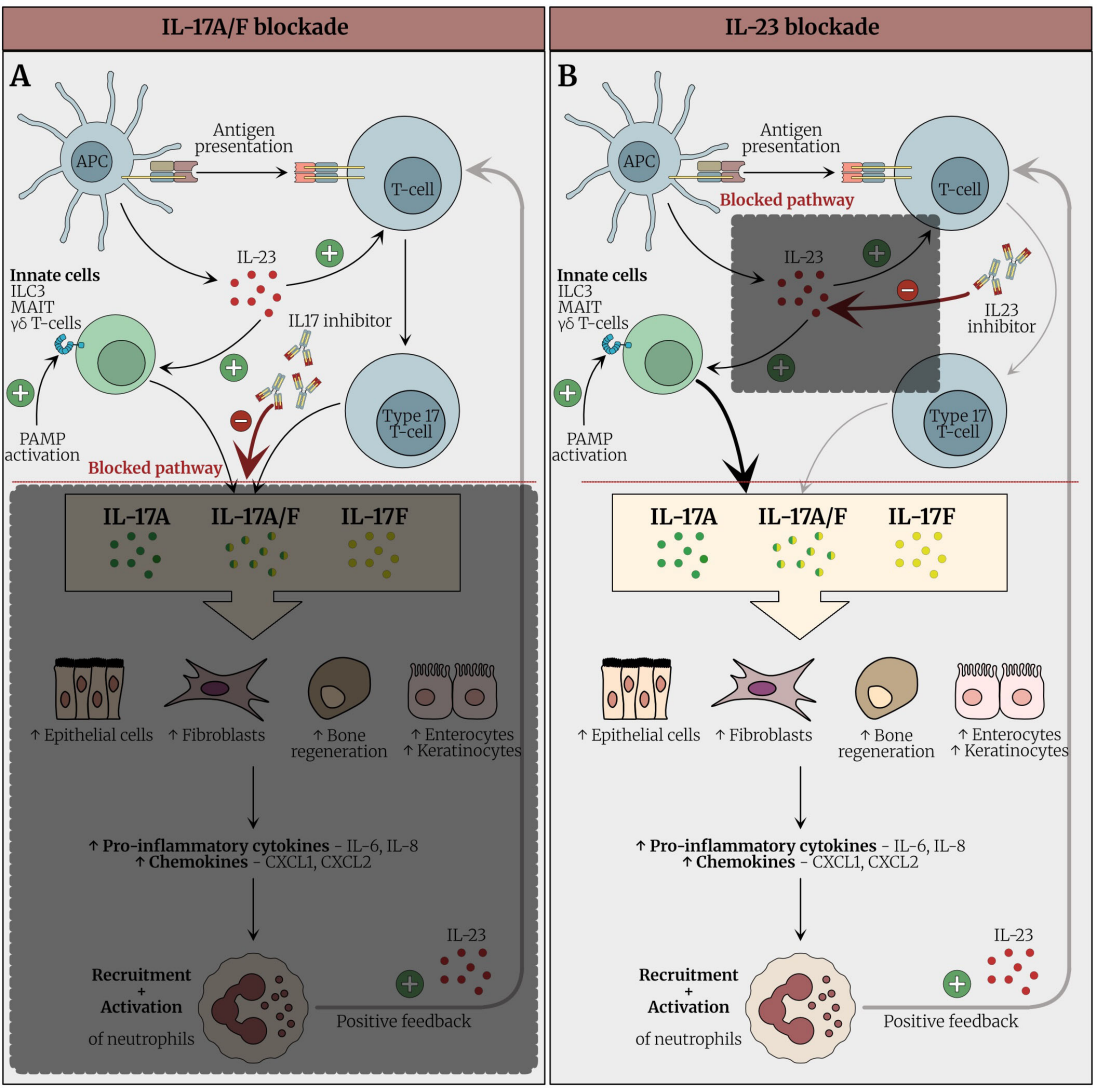
IL-23/17 axis inhibition in Behcet’s syndrome. **(A)** The normal immune response is initiated by the interaction between antigen-presenting cells (APCs) and naïve CD4^+^ T cells, resulting in the differentiation of T-cells into type 17 T cells and expansion of CD8 IL-17 family producing T-cells, driven by IL-23 secreted from the APCs. Various innate immune cells including ILC3 (Type 3 Innate Lymphoid Cells), γδ T-cells, MAITs (Mucosal-Associated Invariant T Cells) and others may also produce IL-17 family cytokines following engagement with IL-23. Furthermore, innate cells in particular may produce IL-17A and IL-17F completely independently of IL-23 stimulation by pattern recognition receptor (PAMP) stimulation and the impact of cytokines including IL-1. Activated neutrophils secrete IL-23, serving not merely as downstream effectors, but as active amplifiers of the immune response via IL-23 secretion. IL-23 has historically been viewed to act upstream of IL-17 by supporting the differentiation and maintenance of type 17 T cells which are responsible for IL-17 secretion. However, neutrophils the key effector innate immune cell are “downstream” of T-cells yet these cells are abundant producers of IL-23. Thus, IL-23 inhibitors prevent the development of type 17 T cells, thereby reducing the production of IL-17 family cytokines (including IL-17A, IL-17A/F, and IL-17F) and attenuating immune activation. However, a combination of high level of IL-23 production by neutrophils and IL-17A and IL-17F production that is independent of IL-23 could account for therapy differences between the IL-23 and IL-17 axis. **(B)** IL-17 inhibition may given more complete neutralization of neutrophil pathway activation compared to IL-23 inhibitor drugs. This concept has been proven in HS, a neutrophilic disorder where anti IL-17 works but anti IL-23 does not. IL-17A inhibitors (e.g., secukinumab and ixekizumab) selectively block IL-17A and IL-17A/F (due to the IL-17A part); however, IL-17F remains active and continues to mediate immune activation, the significance of which remains unclear in Behcet’s syndrome. *IL-17, Interleukin-17; IL-23, Interleukin-23; IL-17i, Interleukin-17*.

In BS, various subsets of T cells have been shown to produce both IL−17 and IFN−γ following prolonged Th17-polarizing stimulation. This dual cytokine response, particularly the unexpected increase in IFN−γ, may reflect enhanced Th1 activity in parallel with Th17 activation, possibly contributing to early neutrophil infiltration and sustained adaptive immune responses in BS (75). Ekinci et al. reported that the Th17/IL-17 pathway is active in BS patients and plays an important role, particularly during acute attacks of the disease (76). A recent meta-analysis revealed significantly higher circulating IL-17 levels in BS patients (33). In a study investigating the immunological role of ERAP1 in BS using a mouse model with incomplete ERAP1 expression, IL-17 was the only factor that showed a significant difference between ERAP1+/− BS mice and WT BS mice. The authors concluded that defective ERAP1 expression plays an important role in BS development through inappropriate regulation of Th17 responses (77). Altogether, this evidence emphasizes the importance of the IL-23/IL-17 axis cytokines in the pathogenesis of BS and constitutes the basis for investigating the therapeutic role of IL-17 and IL-23 inhibitors in the treatment of BS (**Figure 4**).

## The clinical evidence of IL-17 inhibition in BS

Given this impressive amount of immunology and immunogenetics pointing toward the IL-23/17 axis in BS, what is the evidence for efficacy in antagonizing these pathways directly in BS? The clinical effects of secukinumab, a monoclonal anti-IL-17A antibody, have been studied in several manifestations of BS including mucocutaneous, articular and ocular (78–80). One case reported the efficacy of secukinumab in parenchymal neuro-Behçet’s disease (81). **Table 1** summarizes the studies that examined the effect of IL-17 and IL-23 blockade in BS.

**Table 1:** The effect of IL-17 and IL-23 blockade in BS.

Drug	Mode of action	Indication	Study type	Study group	Control group	Intervention	Primary outcome	Limitations	Efficacy
Secukinumab (78)	Anti-IL-17A	Mucocutaneous and articular	Retrospective	5 BS (1 with concurrent AS)	None	Secukinumab 150 mg/4 weeks for patients with concomitant AS and 300 mg4/weeks for PsA	Complete response: decrease of ≥50% of oral ulcers, BASDAI < 4, ASDAS < 1.4 and decrease of ≥50% in BDCAF	Small number of patients,	Effective, with 300 mg/4weeks more effective than 150 mg/4weeks
Secukinumab (79)	Anti-IL-17A	Mucocutaneous and articular	Retrospective	15 BS patients Refractory to colchicine, cDMARDs and at least one anti-TNFα	None	Secukinumab 300 mg/4 weeks for patients with polyarticular disease while the rest received 150 mg/4weeks. Dose increments and/or interval shortening were allowed	Complete response: complete mucosal (no oral ulcers in previous 28 days) and articular control (DAS-28 ≤ 2.6)	Concomitant SpA as the indication for secukinumab,	effective and safe for the long-term treatment of patients with refractory mucosal and articular involvement, with 300 mg every 4 weeks being superior to 150 mg every 4 weeks
Secukinumab (80)	Anti-IL-17A	Ocular	Randomized controlled trial	118 adult patients with active or quiescent intermediate, posterior or panuveitis who have experienced ≥2 episodes of recurrent uveitis in the study eye within the previous 6 months	Placebo, in conjunction to standard of care immunosuppressive therapy	Secukinumab 300 mg every 2 weeks or Secukinumab 300 mg every 4 weeks, in conjunction to standard of care immunosuppressive therapy	reduction in uveitis recurrence or vitreous haze score while withdrawing immunosuppressive medication	complete response definition	no statistically significant differences in uveitis recurrence between treatment and placebo, but Secukinumab showed significant reduction in the mean total post-baseline ISM score compared to placebo
Secukinumab (81)	Anti-IL-17A	Neurologic	Case reports (2)		None	Secukinumab 150 mg/4weeks	Complete resolution of neurologic symptoms		Effective
Ustekinumab (82)	Anti IL-12 and IL-23	Mucocutaneous	Prospective, open-label	14 patients with active oral ulcers refractory to colchicine. Some patients have previously received TNFα inhibitors, apremilast and cDMARDs.	None	90 mg SC at baseline, week 4, and every 12 weeks (45 mg for 3 patients <55 kg)	Complete response: no oral ulcers at week 12	Small sample size, open-label design, no control group, 3 patients on reduced dose	Significant reduction in oral ulcers (64% achieved complete response, 21% partial response). 14% non-responders at week 12. Significant reduction in BSAS score and steroid use. 28% of patients relapsed.
Ustekinumab (83)	Anti IL-12 and IL-23	Mucocutaneous	Prospective, open-label	30 patients with active oral ulcers refractory to colchicine. Patients may have received previously cDMARDs but not bDMARDs.	None	90 mg SC at baseline, week 4, and every 12 weeks	Complete response: no oral ulcers at week 12 and 24	Open-label design, no placebo group, limited sample size	Significant reduction in oral ulcers (60% achieved complete response at week 12 and 95% at week 36). Significant reduction in BSAS score and steroid use. No serious adverse effects.
Ustekinumab (84)	Anti IL-12 and IL-23	Mucocutaneous	Prospective, open-label, phase 2 trial	15 patients with recurrent oral ulcers resistant to colchicine	None	90 mg SC at weeks 0, 4, and 16, with additional injections at weeks 28 and 40 for responders	Reduction in oral ulcer count and pain	Open-label design, small sample size, no control group	Significant reduction in oral ulcers (60% achieved complete response at week 24, 90.9% at week 52). Quality of life and disease activity scores improved. One patient had early relapse. No treatment discontinuation due to adverse effects.
Ustekinumab (85)	Anti IL-12 and IL-23	Mucocutaneous, ocular (anterior uveitis), arthritis and gastrointestinal symptoms (terminal ileum ulcers).	Case report	1 female patient with combined BS, psoriasis and HS. Her symptoms were refractory to colchicine, ocular steroids and cyclosporine	None	45 mg SC at weeks 0, 4, and every 12 weeks	Complete remission of BS and HS symptoms	Single case report, no control group	BS: Complete remission of BS and HS symptoms with no adjunctive immunosuppressive treatment for 36 months. Psoriasis: 50% clinical improvement (PASI50) within 4 weeks.
Guselkumab (86)	Anti IL-23	Mucocutaneous and articular	Retrospective, multicentric observational study	18 patients with refractory mucocutaneous and articular manifestations	None	100 mg SC at weeks 0, 4, then every 8 weeks	Response at week 12, defined as complete: no oral ulcers, tender and swollen joint count of 0, normal CRP. Partial: >50% reduction in oral ulcers and symptomatic joint counts with normal CRP.	Retrospective design, no control group, small sample size, potential bias in selection	55.6% overall response at week 12 (22.2% complete, 33.4% partial). Significant reduction in oral ulcers, tender joints, and CRP. Steroid-sparing effects in 4 patients. No serious adverse events, but 16.7% had end-of-dose effect. Relapses occurred in 2.

IL, Interleukin; BS, Behçet’s disease; AS, Ankylosing spondylitis; PsA, Psoriatic arthritis; BASDAI, Bath Ankylosing Spondylitis Disease Activity Index; ASDAS, Ankylosing Spondylitis Disease Activity Score; BDCAF, Behçet’s Disease Current Activity Form; cDMARDs, Conventional Disease-Modifying Antirheumatic Drugs; TNFα, Tumor Necrosis Factor-alpha; DAS-28, Disease Activity Score-28; ISM, Immunosuppression; SC, subcutaneously; BSAS, Behçet’s Syndrome Activity Score; bDMARDs, Biologic Disease-Modifying Antirheumatic Drugs; HS, Hidradenitis Suppurativa; PASI, Psoriasis Area Severity Index; PASI50, Psoriasis Area Severity Index 50% improvement; CRP, C-reactive protein.

### Mucocutaneous and articular manifestations

Two retrospective studies examined the efficacy and safety of secukinumab in mucocutaneous and articular manifestations. Di Scala et al. conducted a retrospective study evaluating five patients diagnosed with both SpA and BS, all of whom were unresponsive to colchicine, cDMARDs, and at least one anti-TNFα agent. Treatment was administered with secukinumab 150 mg or 300 mg every four weeks, with the higher dose exhibiting greater efficacy. While secukinumab appeared to provide relief for mucocutaneous manifestations, the study’s small sample size and retrospective nature, along with the exclusive inclusion of patients with concomitant SpA, limit the applicability of these findings to the broader BS population. Additionally, the study’s assessment of oral ulceration at isolated time points rather than evaluating cumulative ulcer burden poses a methodological limitation, potentially impacting the interpretation of therapeutic efficacy (78).

A multicenter retrospective study by Fagni et al. investigated 15 patients with mucocutaneous and articular BS who were unresponsive to standard treatments. Administration of secukinumab resulted in response rates of 66.7% at 3 months, 86.7% at 6 months, and 100% by 24 months, with the higher dose (300 mg every 4 weeks) demonstrating superior efficacy. Interestingly, the study also reported improvements in intestinal involvement, a finding that contrasts with the documented risk of IL-17 blockade exacerbating disease activity in IBD. However, despite these encouraging outcomes, certain methodological limitations persist, including a small sample size, potential survival bias, and ambiguity in defining clinically relevant disease activity thresholds, which may impact the broader applicability of these findings (79). However, until randomized controlled trials (RCTs) become available, especially in the era where other agents with stronger evidence that comes from RCTs such as anti-TNFα antibodies and apremilast are available (18, 87), secukinumab should be used cautiously and judiciously in refractory cases failing other approved effective drugs.

### Ocular manifestations

Ocular involvement in BS is common and, if left untreated or undertreated, can lead to blindness. Posterior uveitis or panuveitis accompanied by obliterative retinal vasculitis represents the most common ocular manifestation (88). Despite the efficacy of anti-TNFα in the treatment of BS-related uveitis (6, 9, 13, 16), some patients fail these drugs, emphasizing the need for other therapeutic options. The SHIELD trial was a phase III, randomized, placebo-controlled study that specifically enrolled 118 patients with BS presenting with intermediate, posterior, or panuveitis. The primary endpoint was the prevention of ocular exacerbations, which was not met. However, secukinumab treatment was associated with a significant reduction in concomitant immunosuppressive medication use, suggesting a potential steroid-sparing effect (80). Despite initial optimism, the trial’s primary endpoint—reduction in uveitis recurrence or vitreous haze score during immunosuppressive tapering—did not show a statistically significant difference between secukinumab and placebo, rendering the overall findings underwhelming. However, a notable reduction in the composite immunosuppressive medication (ISM) score in the secukinumab-treated groups suggested a potential steroid-sparing effect, an aspect that warrants further scrutiny. Given that the study population predominantly consisted of severe, treatment-refractory uveitis cases already on immunosuppressive agents, it remains plausible that secukinumab may hold greater therapeutic promise in milder forms of BS-associated uveitis, meriting further exploration in appropriately stratified cohorts.

### Neurologic manifestations

Liu et al. (81) reported two cases of parenchymal neuro-Behçet successfully treated with secukinumab 150 mg every four weeks (81). The first case involved a 27-year-old male with multiple cerebral and cerebellar lesions, oral ulcers, fever, and worsening seizures despite anti-epileptic therapy. After declining glucocorticoids, cytotoxic agents, and anti-TNFα, he was treated with secukinumab, leading to complete neurological resolution. The second case was a 26-year-old male with oral/genital ulcers, periodic fever, and SpA (sacroiliitis, HLA-B*27 positive). His neurological symptoms worsened upon glucocorticoid tapering, with new brainstem and corona radiata lesions emerging. Secukinumab therapy resulted in full symptom resolution, despite the patient being ineligible for anti-TNFα due to latent tuberculosis. While these cases suggest a potential role for secukinumab in parenchymal neuro-Behçet, its efficacy remains unconfirmed in RCTs.

### Vascular manifestations

The efficacy of secukinumab in vascular BS (VBS) was not studied. Reviewing the literature did not reveal any case reports addressing the efficacy of secukinumab in clinical cases of VBS. Moreover, in the SHEILD study, one patient in the secukinumab 300 mg every 4 weeks group died because of a thromboembolic event that was considered not related to study medication (80).

### Gastrointestinal manifestations

Thus far, no studies have directly examined the role of IL-17 inhibition in gastrointestinal manifestations in BS. In their study examining the role of secukinumab in mucocutaneous BS, Fangi and colleagues showed that secukinumab has a beneficial effect in four patients with intestinal involvement as demonstrated by improvement in symptoms and reduction or normalization of fecal calprotectin as described earlier (79). This is in stark contrast to IBD where anti-IL-17 strategies were associated with disease flares. However, Sun and colleagues reported a case of gastrointestinal BS flare-up after secukinumab therapy (89).

A small open-label case series evaluated tofacitinib in thirteen patients with refractory BS, including six with gastrointestinal involvement. While vascular and articular manifestations responded favorably, intestinal outcomes were less consistent. Only one patient achieved mucosal healing, whereas others had persistent ulceration, and one patient discontinued treatment due to clinical worsening (90). These limited findings suggest that JAK inhibition may have a restricted role in gastrointestinal BS, echoing prior observations in Crohn’s disease, where tofacitinib failed to meet primary efficacy endpoints in two phase IIb RCTs (91).

Recent mechanistic data may help contextualize these observations. Giryes and colleagues demonstrated that JAK1 and TYK2 inhibition can paradoxically enhance IL-23 and TNF-α secretion by monocytes through disruption of IL-10–dependent regulatory circuits. This effect, particularly relevant in mucosal environments, may underlie intestinal flares or limited treatment responses seen with JAK inhibitors in susceptible individuals (92).

## The clinical evidence of IL-12/IL-23 inhibition in BS

Ustekinumab is a human monoclonal antibody targeting the p40 subunit shared by IL-12 and IL-23, which has demonstrated promising efficacy in managing refractory mucocutaneous manifestations of BS (93). Baerveldt et al. described the case of a 39-year-old woman with psoriasis, hidradenitis suppurativa and BS who was treated with ustekinumab and demonstrated 50% improvement in psoriasis and complete remission of BS over 36 months (85).

Two prospective studies assessed ustekinumab in refractory mucocutaneous BS (82, 83). In the first, Mirouse et al. treated 14 adults meeting International Study Group for Behçet’s Disease criteria (ISG) with ustekinumab 90 mg at week 0, 4, and then every 12 weeks (82). At week 12, complete remission of oral ulcers occurred in 9 patients (64.2%) and partial remission in 3 (21.4%). Improvements were also noted in disease activity, genital ulcers, steroid dose, arthralgia, and scleritis, while skin lesions resolved in 2 of 4 cases. One patient had persistent intestinal involvement. The second, open-label multicenter study included 30 BS patients with colchicine-refractory oral ulcers (83). At week 12, 18 (60%) achieved complete remission and 9 (30%) partial remission. Improvements were also observed in genital ulcers, arthralgia, pseudo-folliculitis, and overall disease activity (BSAS). Limitations included the open-label design, small sample size, and absence of a control group. In both studies, ustekinumab was generally safe, with headache, nausea, diarrhea, and injection site reactions most frequent.

The prospective phase 2 open-label STELABEC trial investigated the effectiveness and safety of ustekinumab in 15 BS patients, particularly those with recurrent oral ulcers without prior colchicine responsiveness (84). Participants received subcutaneous ustekinumab 90 mg at weeks 0, 4, and 16, with additional doses at weeks 28 and 40 in responders. The trial showed significant improvements in disease activity and quality of life scores at both week 24 and week 52. By week 52, 90.9% of patients maintained their treatment response, with most patients achieving complete responses without treatment discontinuations due to adverse events, highlighting again the safety profile of ustekinumab. These findings provide promising evidence for ustekinumab as a viable treatment option for patients with refractory oral ulcers in BS. The observed improvements in other disease manifestations also suggest potential benefits beyond oral ulceration. It should be emphasized that ustekinumab dose used in IBD, where neutrophils are abundant in the gut, is higher than doses used in these studies, possibly explaining the partial improvement observed in BS patients.

## The clinical evidence of IL-23 inhibition in BS

Guselkumab, a monoclonal antibody targeting IL-23, was evaluated in a retrospective, multicenter study assessing its efficacy and safety in 18 patients with refractory mucocutaneous and articular manifestations of BS (86). All patients were refractory to colchicine, and 15 (83.3%) had previously received at least one other csDMARD or bDMARD. Participants received guselkumab 100 mg subcutaneously at weeks 0 and 4, followed by administration every 8 weeks with 48 weeks follow up. The primary efficacy endpoint was the proportion of responders at week 12, defined as complete (no oral ulcers in the previous 4 weeks, tender and swollen joint counts of 0, and negative C-reactive protein [CRP] level) or partial (at least a 50% reduction in oral ulcers and symptomatic joint counts with a negative CRP level).

After 12 weeks of treatment, 4 (22.2%) patients achieved a complete response, while 6 (33.4%) achieved a partial response. Eight patients discontinued guselkumab treatment: four due to lack of response (refractory arthralgia, oral ulcers, and pyoderma gangrenosum), three due to disease flares (arthralgia, uveitis and inflammatory central nervous system), and one due to an adverse event. The authors concluded that guselkumab may be effective and safe for the treatment of refractory mucocutaneous and articular manifestations of BS up to 48 weeks of follow-up (86).

**Figure 5:**
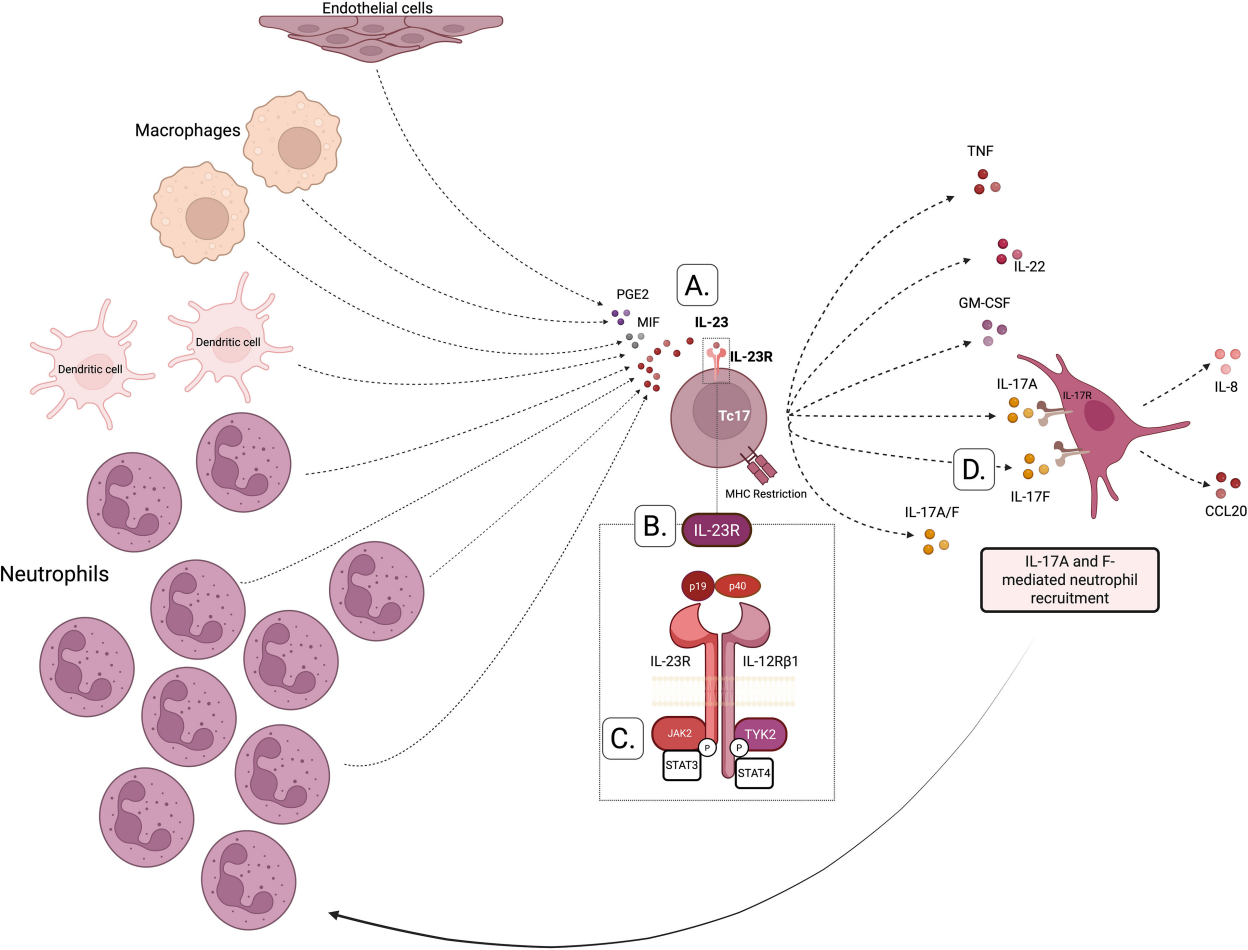
Potential therapeutic bottlenecks in the IL-23/IL-17 pathway in Behçet’s syndrome. **(A)** In BS lesions it is anticipated that abundant neutrophil production of IL-23 that has a positive feedback loop with type 17 cells may be difficult to therapeutically overcome with conventional dosing strategies for anti-IL-23. This scenario may be reminiscent of hidradenitis supportiva (HS) where IL-23 blockers are not effective. **(B)** The IL-23R on T cells forms a potential therapeutic bottleneck as the receptor is only expressed on a subpopulation of T cells and thus may be easier to antagonize the IL-23 pathway. However, for A) and B) if there is also Tc17 activation independent of IL-23 such a scenario of IL-23 blockade may be limited but there is little research exploring these scenarios. **(C)** Blocking Tyk2 or JAK2 signaling also captures the IL-23R bottleneck and several agents are available. However, such therapy could also block IL-10 signaling that also uses this path. **(D)** Blockade of IL-17A and F is the 4^th^ strategy which both blocks effector phase of T-cell activation and also feedback loops whereby IL-17 recruits and activates neutrophils.

Given these somewhat mixed results, we theorize that the large numbers of neutrophils and their ability to produce IL-23 may hinder the ability of anti-IL-23 therapy to neutralize the available IL-23 cytokine. Strategies to overcome this include higher dosage of anti-IL-23 or alternative strategies including targeting of the IL-23 receptor that has a low density of expression on a more limited number of cells. Another strategy may include TYK2 blockade which blocks downstream IL-23 receptor signaling but it must be pointed out that this strategy will also block TYK2-dependent IL-10 pathway signaling (94). This could be detrimental in the intestine where TYK2 blockade has already failed (95), see **Figure 5**.

## Paradoxical effect of IL-17 inhibition

Although IL-17 is involved in the pathogenesis of BS and IL-17 inhibition was studied in the treatment of some manifestations of BS with some promising results, twelve reports in the literature were recently published describing the development of Behçet’s-like syndrome manifestations secondary to IL-17 inhibitors. Indeed, paradoxical effects of secukinumab have already been reported among patients with ankylosing spondylitis (AS) who developed psoriasis after the initiation of secukinumab (96). Herein, we describe an additional case, from our cohort, of a 44-year-old male with AS who failed several anti-TNFα inhibitors and was started on secukinumab 150 mg every four weeks after an induction phase of 150 mg every week for 5 weeks. Three months later he developed oral ulcers and palmoplantar pustulosis on both palms that did not respond to local glucocorticoids. Four weeks later he developed superficial vein thrombosis in the left shin along with panuveitis of the left eye. The secukinumab was stopped and prednisone 1 mg/kg was initiated with subsequent resolution of all manifestations. HLA-B*51 positivity was confirmed.

Analogous to our case, most of the reported cases of Behçet’s-like syndrome developed among patients treated with secukinumab for psoriasis or AS, but two cases developed after ixekizumab treatment (97–106). **Table 2** summarizes the main clinical features of reported cases. Interestingly, all cases developed oral and/or genital ulcers, one patient developed arthritis, two patients had uveitis, two patients had gastrointestinal manifestations, and one patient had bilateral superficial thrombophlebitis. Fever was a common manifestation and was reported in 6 cases (50%). Skin involvement was also common and reported among 8 patients. HLA-B*51 testing was performed in 5 patients and was positive in 4. Secukinumab or ixekizumab withdrawal and glucocorticoids administration were the most common therapeutic actions taken with significant improvement or complete resolution of signs and symptoms. Other therapeutic options included colchicine, apremilast, infliximab and certolizumab pegol.

**Table 2:** Characteristics of reported IL-17 inhibitor-associated Behçet’s-like disease cases.

#	Sex	Age	IL17i agent used	Indication	Symptoms						HLA-B51	Pathergy	Management and outcome	Ref.
					Oral or genital ulcers	MSK	Ocular	GI	Vascular	Other				
1	Male	56	Secukinumab	Psoriasis	Oral	–	–	Abdominal pain	–	Fever, genital folliculitis	–	NA	Management: Secukinumab withdrawal + oral corticosteroids Outcome: Regression	Shiga H. et al., 2017 (97)
2	Male	34	Secukinumab	Ankylosing spondylitis	Both	Knee arthritis	–	–	–	Fever	Positive	NA	Management: Secukinumab withdrawal + oral corticosteroids + certolizumab Outcome: Regression	Dincses E. et al., 2019 (98)
3	Male	29	Secukinumab	Ankylosing spondylitis	Both	–	Bilateral pan-uveitis	–	Bilateral lower extremity thrombo-phlebitis	Fever	–	NA	Management: Secukinumab withdrawal + methylprednisolone and infliximab Outcome: Regression	Dincses E.et al., 2019 (98)
4	Female	46	Secukinumab	Psoriasis	Both	–	–	–	–	Fever, nodular erythematous painful lesions on lower limbs	Positive	Positive	Management: Secukinumab withdrawal + oral corticosteroids Outcome: Improvement	Barrado-Solís N. et al., 2020 (99)
5	Male	48	Secukinumab	Psoriasis	Oral and perianal ulcers	–	Uveitis (type not specified)	–	–	Painful subcutaneous nodular and papulopustules on limbs and back	–	–	Management: Secukinumab withdrawal + oral corticosteroids Outcome: Improvement	Barrado-Solís N. et al., 2020 (99)
6	Female	45	Secukinumab	Psoriasis	Both	–	–	–	–	Diffuse papulopustular eruptions (trunk and extremities)	Positive	NA	Management: Secukinumab withdrawal + oral corticosteroids and apremilast Outcome: Regression	Calleja Algarra A. et al., 2021 (100)
7	Female	46	Ixekizumab	Psoriasis	Both	–	–	–	–	–	NA	NA	Management: Ixekizumab withdrawal + topical corticosteroids Outcome: Complete resolution	Chen D. et al., 2022 (101)
8	Male	43	Secukinumab	Hidradenitis suppurtiva	Both	–	–	–	–	Painful ulcerations on axillary folds, pustules and papules on sternum, back, legs and buttocks	Positive	–	Management: Secukinumab withdrawal + colchicine, topical corticosteroids and IV antibiotics Outcome: Improvement	Avci C. et al., 2023 (102)
9	Female	53	Secukinumab	Ankylosing spondylitis	Both	–	–	Extensive ulcers in pharyngolaryngeal, esophageal, gastric and duodenal mucosa.	–	–	NA	NA	Management: Secukinumab withdrawal + corticosteroids Outcome: Complete resolution	Ogasawara M. et al., 2024 (103)
10	Female	28	Secukinumab	Psoriasis	Both	–	–	–	–	Fever, asymptomatic facial eruptions, anorexia, weight loss, fatigue	–	–	Management: Secukinumab withdrawal + corticosteroids Outcome: Improvement	Liu K. et al., 2024 (104)
11	Male	36	Ixekizumab	Psoriasis	Both					Fever, sore throat, red papules at site of injection	NA	NA	Management: Ixekizumab withdrawal + corticosteroids + oral thalidomide Outcome: resolution	Ren YK. et al., 2025 (105)
12	Female	26	Secukinumab	Behcet’s	Both					Severe flares of mucocutaneous disease	Negative	NA	Management: Secukinumab withdrawal + corticosteroids dose increase + oral thalidomide Outcome: Improvement	Goyal A. el al. 2025 (106)

IL17i, Interleukin 17 inhibitor; MSK, musculoskeletal; GI, Gastrointestinal; HLA-B51, Human Leukocyte Antigen B51; Ref., Reference; IV, intravenous.

Interestingly, in one case secukinumab was initiated to treat BS but paradoxically resulted in severe exacerbation of the mucocutaneous manifestations leading to drug discontinuation.

Although one may wonder whether these reported cases represent an extraintestinal silent, subclinical IBD manifestation (a previously described adverse effect of secukinumab), these cases are more compatible with Behçet’s-like syndrome as HLA B*51 was positive in all patients tested, genital ulcers were common, and superficial thrombophlebitis is more typical in BS.

**Figure 6:**
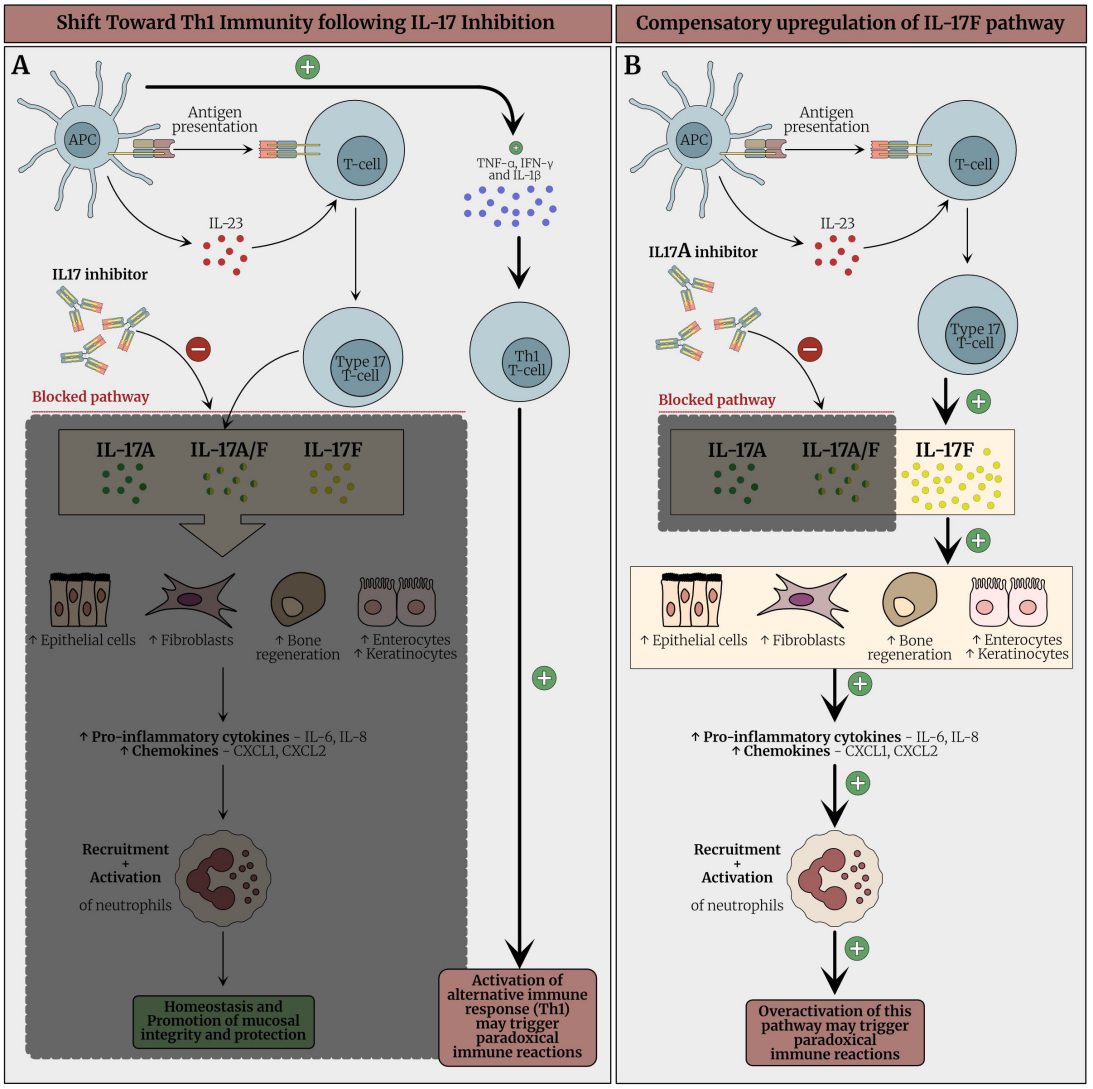
Proposed mechanism underlying paradoxical immune reactions and Behçet-like syndrome following IL-17 inhibition. **(A)** Under physiological conditions, IL-17 produced by Type 17 cells stimulates epithelial cells (keratinocytes and enterocytes, fibroblasts and osteoblasts, and endothelial cells to secrete pro-inflammatory cytokines (e.g., IL-6, IL-8) and chemokines (e.g., CXCL1, CXCL2), which recruit neutrophils to mucosal sites and contribute to mucosal barrier integrity and host defense. Although neutrophils are best known for inflammation they also play key roles in tissue homeostasis and barrier function in the gut and other sites. Reverse translational immunology scheme for how Inhibition of IL-17 disrupts this axis, leading to impaired mucosal immunity and increased susceptibility to mucosal ulceration, particularly in the oral and genital regions. Additionally, IL-17 blockade may result in compensatory upregulation of upstream cytokines such as IL-23 and a shift toward a Th1- immune response. This shift promotes the release of TNF-α, IFN-γ, and IL-1β, activating alternative inflammatory pathways and potentially triggering an exaggerated immune response. These events may culminate in paradoxical autoimmunity, clinically manifesting as Behçet-like syndromes in susceptible individuals. **(B)** IL-17A inhibitors (e.g., secukinumab and ixekizumab) selectively target IL-17A and the IL-17A/F heterodimer, while leaving IL-17F signaling largely unaffected. This selective inhibition may result in compensatory overactivation of the IL-17F pathway, potentially amplifying immune responses and contributing to the emergence of paradoxical autoimmune phenomena via this pathway. IL-17, Interleukin-17; IL-23, Interleukin-23; IL-17i, Interleukin-17; *IL-17i, Interleukin-17 inhibitor; IL-17, Interleukin-17; Th17, T helper 17; IL-6, Interleukin-6; IL-8, Interleukin-8; CXCL1, C-X-C motif chemokine ligand 1; CXCL2, C-X-C motif chemokine ligand 2; IL-23, Interleukin-23; Th1, T helper 1; TNF-α, Tumor necrosis factor alpha; IFN-γ, Interferon gamma; IL-1β, Interleukin-1 beta*.

Possible explanations for the development of Behçet’s-like syndrome cases include the following: 1) effect of IL-17 inhibition on the gut that leads to neutrophil activation, 2) the specific blockade of IL-17A might lead to overproduction of IL-17F, and 3) the switching toward Th1 with subsequent secretion of TNFα and IFNγ. **Figure 6** describes the proposed mechanisms for the development of Behçet’s-like syndrome cases.

## Future directions

As the development of therapeutic options continues, especially for the treatment of diseases within the SpA spectrum, the therapeutic arsenal for BS will ultimately grow and might include the following options: higher loading doses of IL-23 blockers as in IBD, novel molecules like icotrockinra that blocks IL-23R and of course the dual blockade of IL-17A and IL-17F by bimekizumab that showed promising results in AS, PsA and psoriasis (107–109) and also the nanobody including the dual IL-17A/F blocker that is sonelokimab (110). Another therapeutic option that may prove relevant in the future is the combination of biologic DMARDs such as the combination of anti-TNFα and anti-IL-23 or the combination of PDE4 inhibitors and anti-IL-23 inhibitors. The latter combination seems especially and potentially relevant as it blocks IL-23 effects and attenuates the production of IL-23 via neutrophil activation downregulation by PDE inhibitors.

Beyond pharmacologic innovations, it is imperative to recognize that BS exhibits phenotypic heterogeneity aligned with distinct clinical clusters. As delineated in previous sections, IL-23 and IL-17 cytokines likely assume a more central pathogenic role within specific phenotypic subsets, underscoring the plausibility of differential immunologic microenvironments shaping disease expression. Consistent with this perspective, mucocutaneous and articular manifestations may be disproportionately influenced by these cytokines, a premise substantiated by emerging therapeutic data. Consequently, prior to large-scale RCTs evaluating these targeted therapies in BS, it is crucial to refine the delineation of its phenotypic subsets. Moreover, to enhance trial validity and relevance, a paradigm shift is required—eschewing the reliance on outcome measures extrapolated from unrelated diseases in favor of bespoke, phenotype-specific, quantifiable endpoints. In this regard, the conceptual framework established by Hatemi et al. has been instrumental, particularly in characterizing the acne-arthritis-enthesitis cluster (111).

Given that hidradenitis suppurativa (HS) shares extensive neutrophilic inflammatory pathology with BS, it may offer useful insights into future BS therapeutic strategies. Phase II clinical trials of IL-23 inhibitors in HS did not demonstrate clear clinical efficacy, despite evidence that IL-23 is upregulated in lesional tissue (112, 113). This suggests that HS inflammation may, at least in part, be sustained independently of IL-23 signaling, consistent with the disease’s strong neutrophil- and IL-1-dominated innate immune signature. Indeed, IL-1 blockade with agents targeting IL-1β (such as anakinra and lutikizumab) has shown clinical benefit in subsets of HS patients, underscoring the pathogenic importance of IL-1β–driven pathways, whereas IL-1α blockade with bermekimab yielded less consistent results (114). Beyond cytokine signaling, IL-1 also amplifies neutrophil recruitment and activation in HS lesions, driving a self-perpetuating inflammatory loop that may similarly operate in Behçet’s disease.

Behçet’s syndrome may share a similar immunopathogenic architecture: while IL-23 and IL-17 pathways are genetically and immunologically central, the marked neutrophil activation and IL-1-dependent tissue injury likely modulate therapeutic responses. Notably, IL-1 inhibitors have shown partial or variable efficacy in refractory mucocutaneous BS, suggesting that IL-1–driven inflammation may contribute to disease activity in selected patient subsets (115). In this context, the established efficacy of apremilast in BS is of particular interest, as its PDE-4–mediated inhibition of neutrophil activation, ROS production, and NET formation may partly account for its clinical benefit and provides an additional mechanistic rationale for integrating innate-targeted approaches with IL-23 pathway modulation in future studies (55).

## Conclusion

The IL-23/IL-17 axis is increasingly recognized as an important immunological pathway in Behçet’s syndrome, supported by genetic associations, T cell polarization toward Th17/Tc17 subsets, cytokine profiling in affected tissues and related neutrophil biology. While clinical responses to IL-23/17-targeted therapies have been variable across studies, mainly consisting of case reports, case series and retrospective studies, emerging data suggest a differential efficacy profile across BS subtypes. Mucocutaneous and articular manifestations may be more amenable to this approach, whereas ocular and neurological involvement appear less responsive, and in rare cases, paradoxical exacerbations have been reported. These observations highlight the need for better immunophenotypic stratification and mechanistically informed trials to delineate the precise therapeutic window of IL-17/23 inhibition within the heterogeneous clinical spectrum of BS. Finally, although we focus on the IL-23/17 axis in BS that dovetails with both neutrophilic and T-cell biology, it is important to acknowledge that work is needed to understand cytokine heterogeneity across the spectrum of disease and to what degree other pathways including TNF and interferon, for example, dominate the BS phenotype in other cases.
